# The brain adjusts grip forces differently according to gravity and inertia: a parabolic flight experiment

**DOI:** 10.3389/fnint.2015.00007

**Published:** 2015-02-11

**Authors:** Olivier White

**Affiliations:** ^1^Unité de Formation et de Recherche en Sciences et Techniques des Activités Physiques et Sportives, Université de BourgogneDijon, France; ^2^Unit 1093, Cognition, Action, and Sensorimotor Plasticity, Institut National de la Santé et de la Recherche Médicale (INSERM)Dijon, France

**Keywords:** grip force, load force, rhythmic movement, gravity, internal model, inertia

## Abstract

In everyday life, one of the most frequent activities involves accelerating and decelerating an object held in precision grip. In many contexts, humans scale and synchronize their grip force (GF), normal to the finger/object contact, in anticipation of the expected tangential load force (LF), resulting from the combination of the gravitational and the inertial forces. In many contexts, GF and LF are linearly coupled. A few studies have examined how we adjust the parameters–gain and offset–of this linear relationship. However, the question remains open as to how the brain adjusts GF regardless of whether LF is generated by different combinations of weight and inertia. Here, we designed conditions to generate equivalent magnitudes of LF by independently varying mass and movement frequency. In a control experiment, we directly manipulated gravity in parabolic flights, while other factors remained constant. We show with a simple computational approach that, to adjust GF, the brain is sensitive to how LFs are produced at the fingertips. This provides clear evidence that the analysis of the origin of LF is performed centrally, and not only at the periphery.

## Introduction

There is evidence that when holding an object with a precision grip, a minimal grip force (GF, normal to the contact surfaces) must be applied to prevent the object from slipping under the influence of load forces (LF, tangential to the contact surfaces). A normal force generates a proportional friction force between the fingers and the object that assists in stabilizing the grip against external disturbances. Potential slips in the use of erroneous GF adjustments emphasize the need for anticipatory mechanisms that can predict the required GF as a compromise between keeping the object in hand while minimizing muscle fatigue.

One of the most frequent actions we perform everyday involves accelerating and decelerating an object held in precision grip. According to Newton's second law, accelerating an object generates an inertial force. In a gravitational field, the total LF increases when an object is accelerated upwards, while it decreases when the object is accelerated downwards. In a large panel of tasks, humans scale and synchronize their GF in anticipation of the expected LF. For instance, this tight coordination between GF and LF has been shown when transporting objects (Flanagan and Tresilian, 1994), during locomotion (Gysin et al., 2003) or when the load at the fingertips depends on position (Descoins et al., 2006), velocity (Flanagan and Wing, 1997), acceleration (Flanagan et al., 1993; Flanagan and Rao, 1995), and even gravity (Augurelle et al., 2003).

The implementation of anticipatory mechanisms in the Central Nervous System (CNS) has been demonstrated. The brain uses an arm efference copy in conjunction with an internal model of the arm, the object and the environment to anticipate the resulting LF and thereby adjusts GF appropriately (Wolpert et al., 1995; Flanagan and Wing, 1997; Kawato, 1999; Nowak et al., 2007; White et al., 2013). By experiencing object manipulations in many situations, we learn and refine a set of internal models, with each of them suitable–or at least constituting a reasonable first guess–for one or a small set of contexts (Blakemore et al., 1998; Wolpert et al., 1998b). This can explain why we are able to switch between different objects and contexts quickly and effortlessly (White and Diedrichsen, 2013) and suggests that the CNS maintains a set of internal models in memory simultaneously (Wolpert et al., 1998a; Haruno et al., 2001).

However, to our knowledge, only a few studies quantitatively investigated the robustness of GF/LF coordination to external parameters. A strong linear relationship between GF and LF was first reported in rhythmic arm movements by Flanagan and Wing (1995). These authors and others also evaluated the effects of frequency, surface texture, friction and voluntary GF level on the gain and offset that describe the modulation between the two forces (Flanagan and Wing, 1995; Saels et al., 1999; Augurelle et al., 2003). A similar approach demonstrated that gains of the GF/torque relationship increased with smoother objects (Kinoshita et al., 1997). Furthermore, in an interesting paradigm, Zatsiorsky and colleagues investigated the differential effects of gravity and inertia on GF during rhythmic manipulations of hand-held objects (Zatsiorsky et al., 2005). These authors analyzed the relationship between GF and LF in different conditions of masses and accelerations induced to the hand-held load. By analysing these regressions, they concluded that the controller regulates GF in static (holding) and dynamic (moving) tasks differently.

The aim of this study is to further understand quantitatively how contextual parameters such as mass of the object, acceleration and gravity influence GF. We asked participants to rhythmically move eight masses at four frequencies along the vertical axis. First, we verified how the combinations of mass and frequency influenced the gain and the offset of the GF–LF relationship (Zatsiorsky et al., 2005). Second, following the Equivalence Principle stating that local effects induced by gravity and acceleration are identical–physics in an accelerating spacecraft is equivalent to physics in a gravitational field–, does it mean that people adjust GF to gravitational and inertial forces identically? If this hypothesis holds, then participants should not exert different GF against equivalent LF but generated with unequal contributions of mass and acceleration. We tested this hypothesis with a computational model and data recorded in altered gravity.

## Materials and methods

### Subjects

Six adult (25.3 years old, 5F) participated voluntarily in this study. Participants used their preferred hand (four right-handed) and reported no previous history of neuropathies or trauma to the upper extremities. The experimental protocol was approved by the Ethics Committee of the Université catholique de Louvain (Belgium). In addition, 3 new right-handed participants (27, 32 and 31 years old, 1F) were involved in a parabolic flight experiment (37th ESA Parabolic Flight Campaign). Their health was assessed by their National Centers for Aerospace Medicine as meeting the requirement for parabolic flight. No participant reported sensory or motor deficits and none had previously experienced parabolic flight. The procedures were approved by the European Space Agency Safety Committee, by the Université catholique de Louvain ethics committee and by the French CCPPRB (Comités Consultatifs de Protection des Personnes se prêtant à des Recherches Biomédicales).

### Equipment

**Figure 1:**
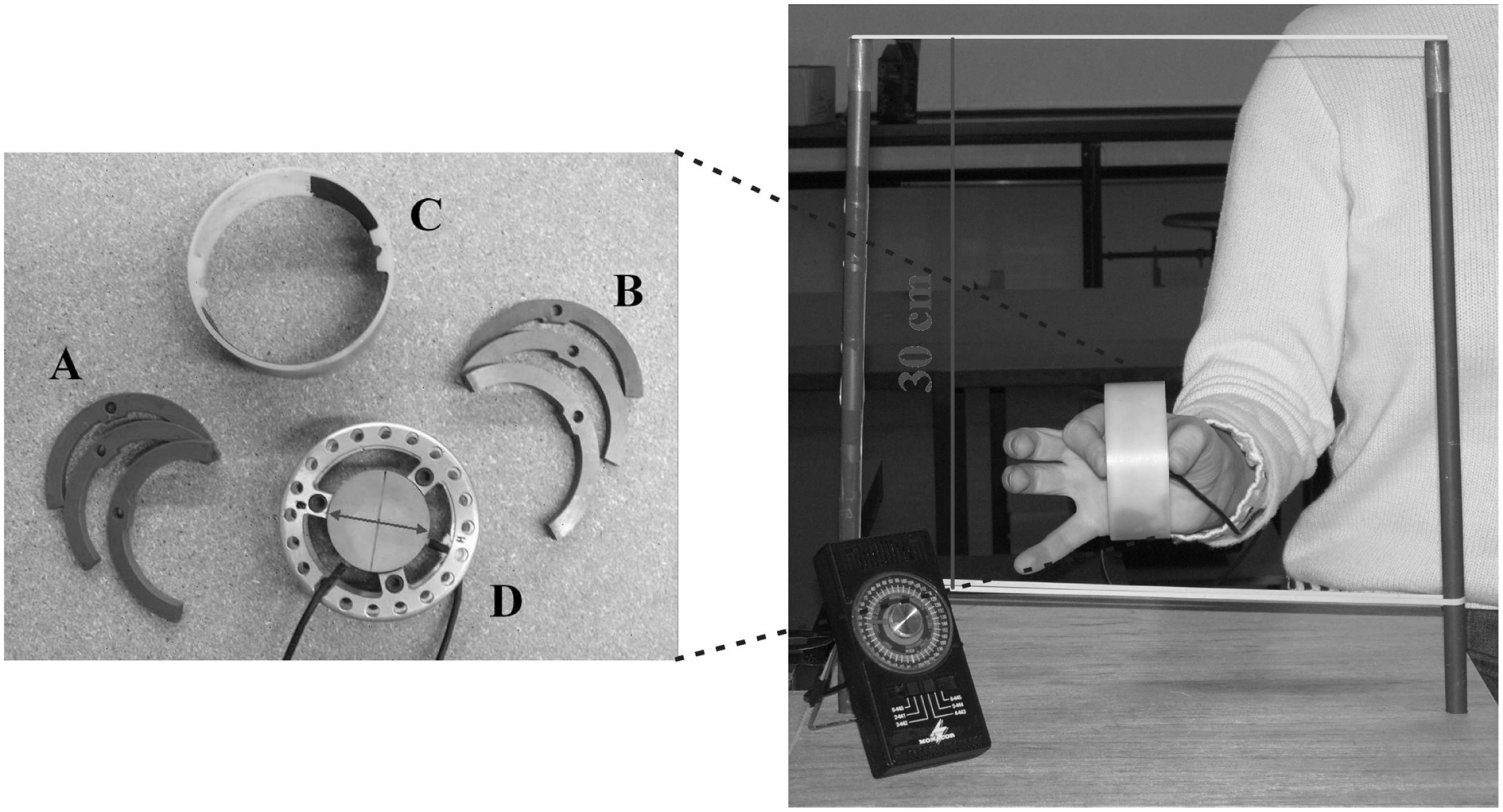
**The manipulandum with half-rings and the set-up**. The light **(A)** and heavy **(B)** half-rings, the opaque cover **(C)** and the manipulandum **(D)** equipped with the sensors. (Right panel) Frontal view of the apparatus held between the thumb on one side and the index on the other side. Arm displacement was limited by two elastic bands spaced 30 cm apart. The black device in the bottom-left corner is the metronome that emitted periodic tones.

GF/LF coupling was studied while holding a cylindrical object with the index finger and the thumb during rhythmic vertical arm movements. The instrumented object (manipulandum) was equipped with two circular grasp surfaces placed on two parallel force sensors. The sensors measured the two tangential force components (F_x_ and F_y_) and the normal force component (F_z_) under the thumb on one side and the index finger on the opposite side. The device was the same as in White et al. (2005) and is technically described in White et al. (2009). The total mass of the object could be varied by inserting half-rings weighting either 8 or 83 g. In addition to the empty manipulandum, a total of seven combinations of six half-rings could generate eight different configurations of masses (see Figure 1 and Table 1).

**Table 1:** **Detailed combinations of half-rings for the eight mass conditions**.

	**Upper half body**	**Lower half body**	**Mass (g)**
M1	Empty	Empty	235
M2	LLL	LLL	283
M3	LLL	LHL	358
M4	LHL	LHL	433
M5	LHL	HLH	508
M6	HLH	HLH	583
M7	HLH	HHH	658
M8	HHH	HHH	733

*Labels “L” and “H” refer to a Light or Heavy half-ring, respectively*.

In order to avoid any rotational slip induced by a torque around the grip axis (Z-axis, normal to the contacting surface), the lower half-body was always heavier than the upper half-body when the condition required an asymmetric half-rings configuration (M3, M5, M7, see Table 1). An opaque cover around the manipulandum kept the masses interlocking and gave no cue to the participant about the hidden distribution. The empty manipulandum and the opaque cover weighted 0.235 kg together (M1 in Table 1).

### Experimental procedure

**Figure 2:**
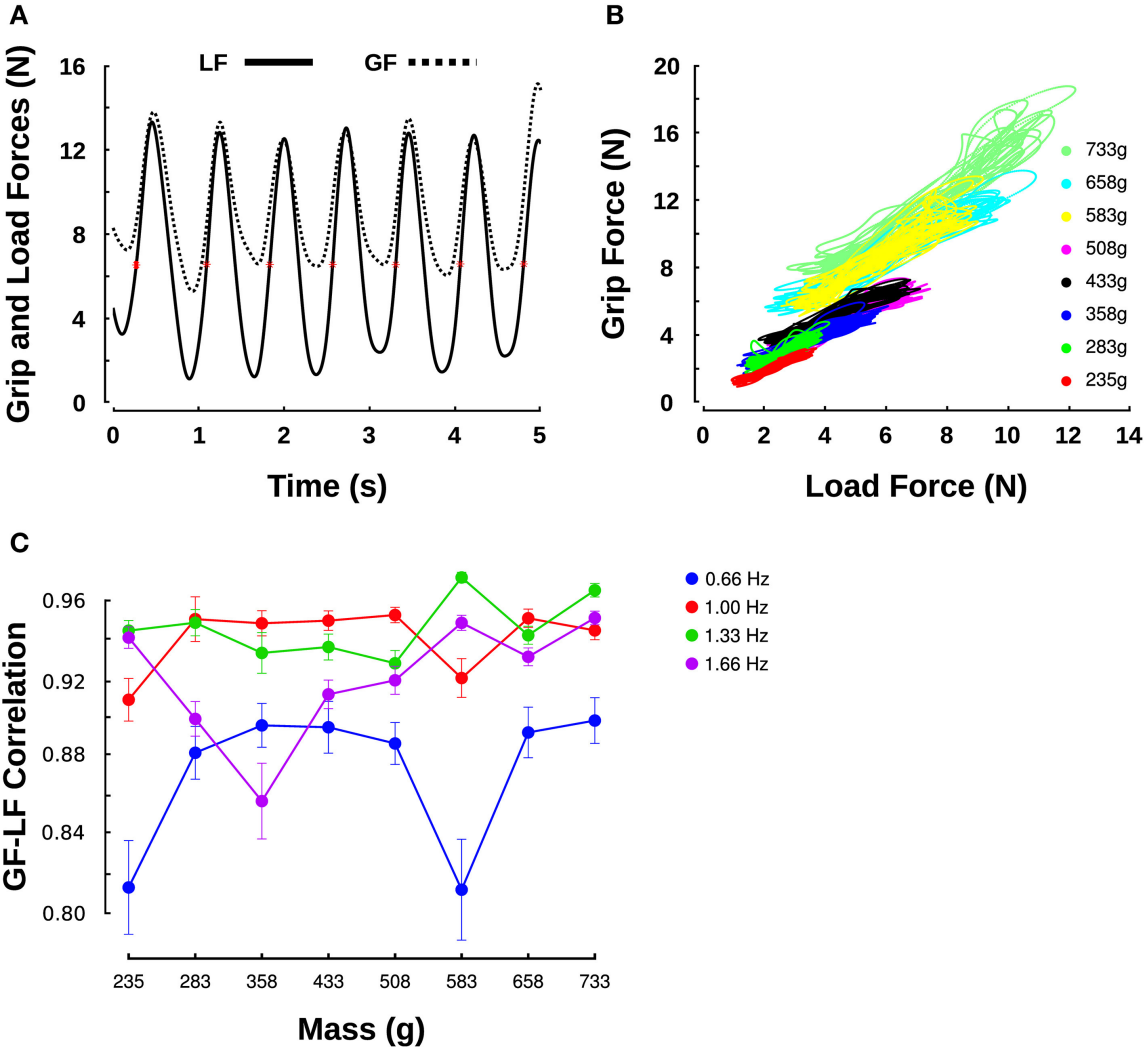
**Grip force/load force relationship across different masses**. **(A)** Evolution of GF (dashed line) and LF (solid line) over time during oscillations at 1.33 Hz with a mass of 0.583 kg. Red asterisks indicate when LF = mg, that is, when a = 0. **(B)** GF-LF relationships for all masses moved at a frequency of 1 Hz, for one participant. **(C)** Correlation coefficients revealing the goodness of linear fits between GF and LF within each movement cycle in all mass (X-axis) and frequency conditions (individual series).

The six participants were comfortably seated in a chair. At a signal from the experimenter, she/he grasped the manipulandum between the thumb and the index finger. Participants were instructed to perform vertical rhythmic arm movements between two horizontal elastic bands 30 cm apart (Figures 1E, 2A). The oscillations were timed by a metronome twice a cycle, at the two extremities of the trajectory. No indication about the magnitude of the load was provided. The vertical position *y*(*t*) over time therefore followed a sine wave according to $y(t)=\frac{A}{2}\sin\left(2\pi ft\right)$ where *A* is the amplitude (30 cm) and *f* the frequency.

One experimental session comprised eight blocks. Each block corresponded to a certain mass configuration (M1–M8 in Table 1). During each block, the participant performed the movement at four different frequencies (0.66, 1, 1.33, and 1.66 Hz) during 30 s (8 masses × 4 frequencies = 32 conditions). The frequencies were randomized within a block and block order was counterbalanced across participants. No specific instruction was provided regarding GF strategy. Five-minute pauses between blocks prevented any fatigue effect.

### Parabolic flight experiment

This part of the experiment took place in the Airbus A300 ZEROg aircraft on two flights from Bordeaux (France). See White et al. (2005) for additional details on the procedure. A single parabolic flight profile generated a sequence of episodes of normal (1 g), hyper (1.8 g), micro (0 g), hyper (1.8 g), and normal (1 g) gravity of about 20 s duration each. Our three participants were involved in another experiment but we dedicated one full parabolic profile at the end of their session to the purpose of this experiment. Participants were therefore accustomed to the new environments, regarding this specific task (Augurelle et al., 2003). We asked each of them to generate fast vertical movements with the same instrumented object as described above (configured with M1) throughout the parabolic profile. Participants performed on average (SD) 45.3 (10.5) cycles in 0 g, 37.7 (12.4) cycles in 1 g and 22 (5) cycles in 1.8 g.

### Data processing

The signals from the transducers and the metronome were digitized on-line at 400 Hz with a 12-bit 6071E analog-to-digital converter in a PXI chassis (National Instruments, Austin, TX). The force applied normal to each grasp surface was calculated as -Fz. The total GF was calculated as the average of the GFs applied by the thumb and the index on each transducer. LF magnitude was computed as: $\text{LF}=\sqrt{{\left({\text{F}}_{\text{x},1}+{\text{F}}_{\text{x},2}\right)}^{2}+{\left({\text{F}}_{\text{y},1}+{\text{F}}_{\text{y},2}\right)}^{2}}$ (for transducers 1 and 2). Grip and LFs were low-pass filtered at 15 Hz (autoregressive dual pass filter). In some trials, low-frequency changes in the GF were observed. Therefore, a high-pass filter at a cutoff frequency of 0.2 Hz was passed to the raw GF and an offset was added such that the filtered force had the same means as the unfiltered data (dual pass fourth order Butterworth filter). We verified that each subjects followed the rhythm dictated by the metronome with a *t*-test comparing performed and target frequencies (all *t*_5_ < 1.9, all *p* > 0.106).

In any constant gravitational environment, an accelerated object generates both a gravitational force (mg) and an inertial force (ma) that yield to a LF tangential to the finger/object interface, LF = mg + ma. The second derivative of *y*(*t*) leads to the acceleration *a*(*t*) = −2*A*π^2^*f*^2^*sin*(2π*ft*). Previous studies reported a robust linear relationship between GF and LF (Flanagan et al., 1993). Therefore, we computed the linear regression GF = αLF + β between GF and LF across each trial of 30 s. The gain α quantifies the amount of extra GF induced by a 1-N increase of LF. The offset β reflects the amount of residual GF when LF is zero. This latter situation is more a theoretical condition on Earth since weight cannot be nullified. It is however possible to counterbalance gravitational and movement accelerations, but only transiently, such that *a* = −*g*. Microgravity environments allow circumventing these limitations.

Finally, an iterative procedure identified a 0.5 N-width interval of LF across the 32 collapsed conditions (8 masses × 4 frequencies), under the constraint that it maximized the number of mass/frequency combinations. The LFs varied between 0 and 18 N, across participants and conditions. The optimal 1 N-bin LF ranged from 3.25 to 3.75 N and covered 28 combinations out of 32 (87.5%). This interval included LF generated by slow movements with a heavy mass and LF induced by fast oscillations with a lighter weight, therefore covering a wide spectrum of parameters. The lightest Mass 1 has been excluded, because LF generated by its movement did not intersect the 3.25–3.75 N interval in all participants. We again calculated the regressions between GF and LF for each mass/frequency conditions.

Quantile-quantile plots were used to assess normality of the data. *T*-tests and repeated measures ANOVAs were performed in Matlab (The Mathworks, Chicago, IL) on gains (α) and offsets (β). Partial eta-squared are reported for significant results to provide indication on effect sizes.

## Results

Figure 2A shows an example of GF and LF over time for a single participant moving a mass of 0.583 kg (M6) at the frequency of 1.33 Hz. The LF (Figure 2A, solid line) followed a sinusoid centered on the object's weight (about 5.7 N, red asterisks). The GF (Figure 2A, dashed line) was synchronized with the LF. The right panel plots the GF-LF relationships for a single participant for every mass and for a given frequency of 1 Hz (Figure 2B). As already observed in previous studies, the scatter plots follow good linear relationship (Flanagan et al., 1993; Saels et al., 1999) which averaged to 0.91 (*SD* = 0.07) overall. Figure 2C reports correlations for the linear fits between GF and LF across masses and for the four frequency conditions. The ANOVA reported no main effect of mass [*F*_(7, 144)_ = 0.8, *p* = 0.591] nor interaction with frequency [*F*_(21, 144)_ = 0.86, *p* = 0.637]. However, frequency significantly influenced the quality of the fit [*F*_(3, 144)_ = 12.5, *p* < 0.001, η^2^_p_ = 0.18]; a *t*-test revealed that both 1 and 1.33 Hz provided significantly better correlation coefficients than the lowest frequency (1 Hz: *t*_5_ = 3.2, *p* = 0.024, η^2^_p_ = 0.67; 1.33 Hz: *t*_5_ = 2.9, *p* = 0.033, η^2^_p_ = 0.63). Although gains look visually comparable across mass conditions, substantial offsets proportional to the mass are qualitatively observed. In other words, the average GF exerted during the movement increased for heavier masses. For instance, an average LF of 2.3 N (0.235 kg) led to an average GF of about 2 N (Figure 2B, dotted lines, red cluster) and an average LF of 5.7 N (0.583 g) led to an average GF of 8.5 N (Figure 2B, dashed-lines, yellow cluster). The next section quantifies gains and offsets of this GF-LF relationship.

### Global effects of acceleration and mass

**Figure 3:**
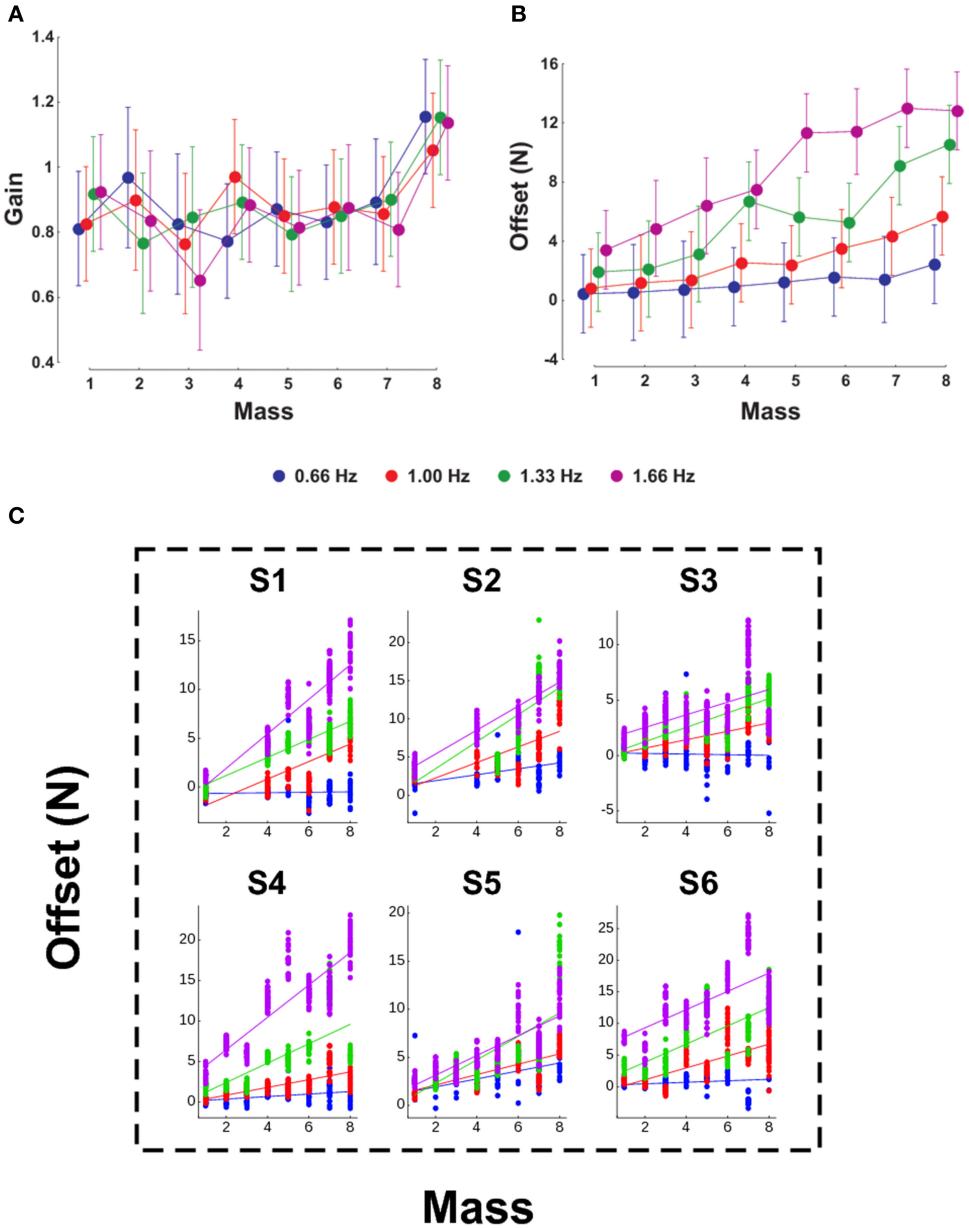
**Influence of mass and frequency on GF-LF linear regression parameters**. The gain **(A)** and offset **(B)** of the regression are presented for the eight masses (X-axis). In each panel, the four plots correspond to the frequencies 0.66, 1, 1.33, and 1.66 Hz. **(C)** Offset in function of mass, as in **(B)**, but in our six individual participants. The average pattern observed in **(B)** is very consistent within participants.

We investigate whether mass and acceleration (through the frequency of oscillations) have an influence on the parameters of the linear regression between GF and LF. We conducted a Two-Way ANOVA on the gain and offset taking the mass as the first factor and the frequency as the second factor. Figure 3 presents the evolution of the gain (Figure 3A) and offset (Figure 3B) for the eight masses (x-axis) and the four frequencies (four plots). Gains were not influenced by frequency [*F*_(3, 144)_ = 0.2, *p* = 0.894] and were stable across the entire mass range except for M8 [*F*_(7, 144)_ = 5.24, *p* < 0.001, η^2^_p_ = 0.19; *t*-test M1–7 vs. M8, *t*_5_ = −2.87, *p* = 0.035, η^2^_p_ = 0.62]. In contrast, the offset was influenced both by mass [*F*_(7, 144)_ = 9.42, *p* < 0.001, η^2^_p_ = 0.18] and frequency [*F*_(3, 144)_ = 42.28, *p* < 0.001, η^2^_p_ = 0.34], but without significant interaction [Figure 3B, *F*_(21, 144)_ = 1.28, *p* = 0.199]. For a given mass below M5, the offsets were not different whereas they significantly diverged with frequency for masses above M5. Interestingly, there was a significant linear correlation between mass and offset in every frequency condition (*r* = 0.2, 0.63, 0.65, and 0.62 for frequencies 0.66, 1, 1.33, and 1.66 Hz, respectively, all *p* < 0.001). The steepness of this relationship increased with frequency (slopes = 0.18, 0.73, 1.14, and 1.41 for frequencies 0.66, 1, 1.33, and 1.66 Hz, respectively). Finally, this pattern was consistent in our six individual participants (Figure 3C).

### Grip force control in a given range of load force

In this section, we tested how the mass of the object and the frequency of movement influenced the parameters α^*^ and β^*^ of the linear regression between GF and LF. The notation overline is used for LF since we assume it is constant within the interval, compared to the whole amplitudes observed during the experiment: GF = α^*^LF + β^*^. The dataset considered here covered 28 combinations of mass and frequency out of 32 (87.5%).

**Figure 4:**
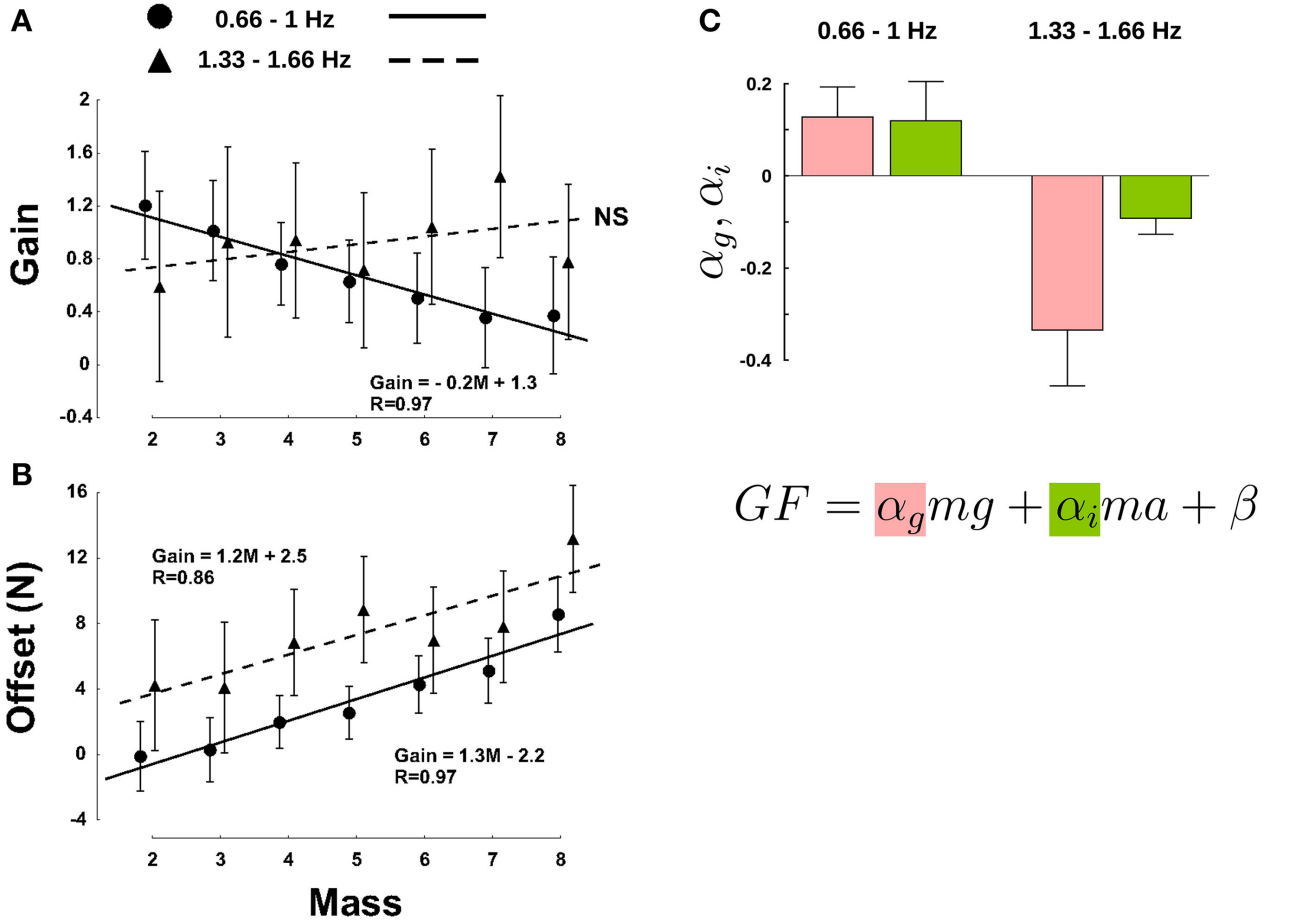
Mass and movement frequencies have different effects on the regulation of GF, as demonstrated by changes in the gain α^*^ (A) and offset β^*^ (B). The masses on the X-axis refer to labels in Table 1 and are shifted by a constant amount of 75 g. Note that M1 was discarded because all frequency conditions generated LF outside the interval. Disks correspond to low frequencies (0.66 and 1 Hz) and triangles correspond to high frequencies (1.33 and 1.66 Hz). **(C)** Gravitational (red bars) and inertial gains (green bars) calculated separately for the low frequencies (two left bars) and for the two high frequencies (two right bars). Error bars are between participants SD. Determination coefficients for the low and high frequency clusters were resp. *R*^2^ = 0.67 and *R*^2^ = 0.62 (range across individual subject fits: *R*^2^ = 0.37–0.9).

Data were split in two clusters upon frequency condition: low frequencies (0.66 and 1 Hz) and high frequencies (1.33 and 1.66 Hz). Overall, the gain was not affected by mass [Figure 4A, *F*_(6, 61)_ = 1.12, *p* = 0.356] nor by frequency condition [*F*_(1, 61)_ = 1.24, *p* = 0.269]. However, there was a significant interaction, *F*_(6, 61)_ = 3.44, *p* = 0.006, η^2^_p_ = 0.23, as gains decreased in the low frequency cluster (Figure 4A, disks) and increased at higher frequencies (Figure 4A, triangles). Furthermore, mass influenced the offset in both slow and fast movements [*F*_(6, 62)_ = 8.37, *p* < 0.001, η^2^_p_ = 0.33], but without interaction, as revealed by the parallel increase in the linear fit [Figure 4B, *F*_(6, 62)_ = 0.32, *p* = 0.926]. Finally, a larger frequency induced an increment of offset, as quantified by a statistically significant main effect on offset, *F*_(1, 62)_ = 36.89, *p* < 0.001, η^2^_p_ = 0.24. Altogether, we show that equivalent LF does not imply similar GF.

The above analysis is still general as it only demonstrates a *global effect* of different contributions of gravitational and inertial forces to the adjustment of GF. We went one step further and tested how these two separate components affected GF for an equivalent LF. To do so, we ran a multiple regression analysis to derive the coefficients of the following equation, F = α_g_mg + α_i_ma + β, where α_g_ is the “gravitational gain,” α_i_ is the “inertial gain” and β is the offset (least squares linear fit). This approach yields to two predictions. If these two components are treated separately, then α_g_ ≠ α_i_, otherwise, α_g_ = α_i_ = α, bringing back the general relationship GF = αLF + β. In both cases, however, the parameter α, that includes both α_g_ and α_g_, is influenced by gravity and acceleration, as shown in Figure 4A. However, we still do not know how.

Figure 4C presents values of gravitational (α_g_) and inertial gains (α_i_) for low (two left bar plots) and high frequencies (two right bar plots). An ANOVA revealed a main effect of frequency [*F*_(1, 20)_ = 16.5, *p* < 0.001, η^2^_p_ = 0.4] and parameter [*F*_(1, 20)_ = 2, *p* = 0.03, η^2^_p_ = 0.1] but no interaction [*F*_(1, 20)_ = 2.29, *p* = 146]. Independent *t*-tests confirmed the observation that the gravitational and inertial force components are adjusted independently [*t*_(10)_ = 2.3, *p* = 0.044, η^2^_p_ = 0.35]. In other words, the same increment of gravitational and inertial force leads to different adjustments of GF.

### Parabolic flight experiment

**Figure 5:**
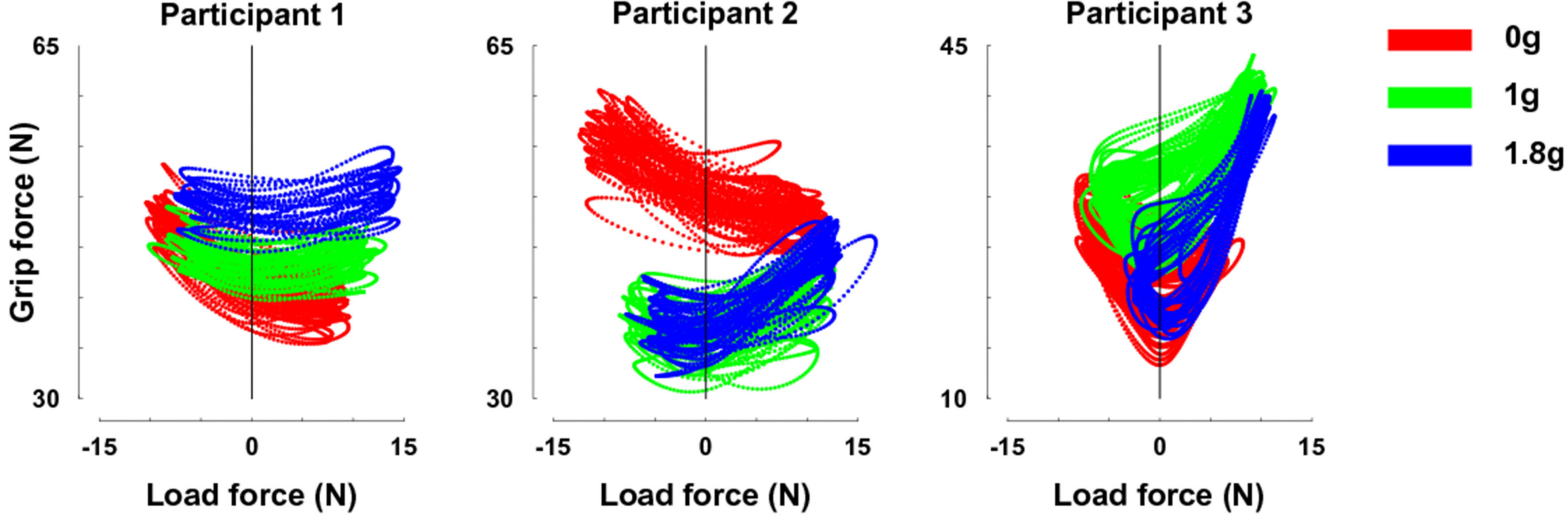
**GF-LF relationships for the three participants in 0 (red trace), 1 (green), and 1.8 g (blue trace)**. Note that y-scales are different but cover the same range of GF. Error bars are between participants SD.

In a control experiment, we had the opportunity to truly change gravity itself. Figure 5 presents relationships between GF and LF for our three separate participants in 0 (red trace), 1 (green), and 1.8 g (blue trace). While LF overlapped across gravitational conditions (Table 2), participants adopted different GF, leading to well identifiable clusters.

**Table 2:** **Individual descriptive statistics for the three subjects across 0, 1, and 1.8 g**.

		**S1**	**S2**	**S3**
GF (N)	0 g	42.61 (30.79–60.22)	51.09 (29.66–70.05)	23.00 (14.58–35.56)
	1 g	43.87 (36.41–59.72)	37.87 (25.64–51.05)	32.39 (18.13–47.49)
	1.8 g	49.39 (39.74–61.43)	39.81 (29.35–53.32)	26.69 (13.74–45.92)
LF (N)	0 g	−0.63 (−11.35–8.91)	−1.94 (−14.06–11.88)	0.88 (−7.67–8.61)
	1 g	0.19 (−10.79–10.75)	1.98 (−8.36–12.64)	2.27 (−6.58–11.53)
	1.8 g	1.52 (−10.67–11.88)	2.80 (−7.95–12.87)	2.34 (−4.55–10.26)

*The three upper rows report mean GF and the three lower rows report mean LF. Range of forces are in brackets (minimum to maximum)*.

**Figure 6:**
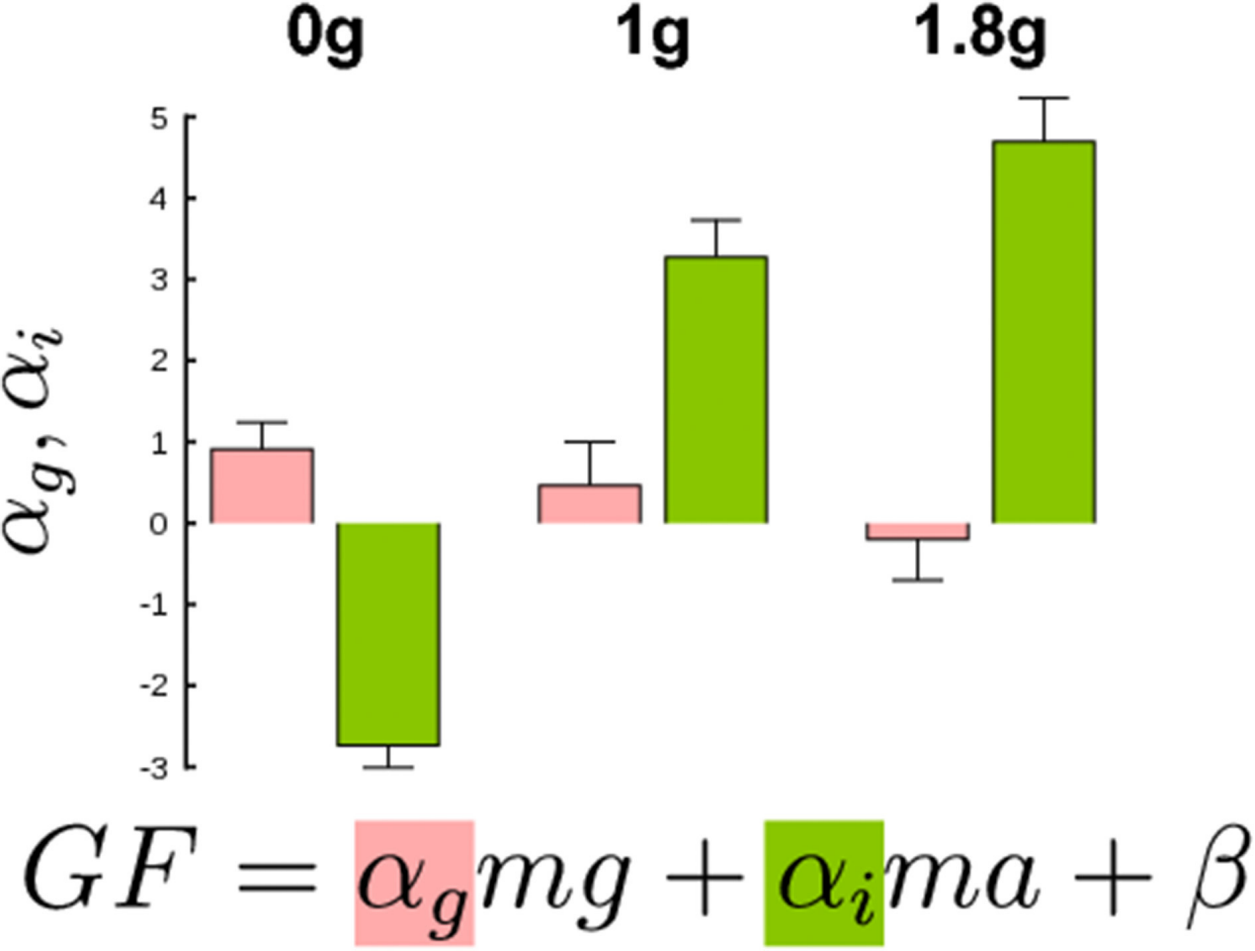
**Gravitational (red bars) and inertial gains (green bars) calculated separately in the three gravitational levels**. Note that gain values are larger than in Figure 4C because participants produced high GF. Error bars are between participants SD. Determination coefficients in 0, 1, and 1.8 g conditions were resp. *R*^2^ = 0.85, *R*^2^ = 0.83, and *R*^2^ = 0.84 (range across individual subject fits: *R*^2^ = 0.31–0.95).

We conducted the same regression analysis as above and calculated the gravitational and inertial gains. Figure 6 presents bar plots of gravitational (α_g_, red) and inertial gains (α_i_, green) for 0, 1, and 1.8 g environments. An ANOVA reported main effects of gravitational environment, type of gain and an interaction between these two factors (all *F* > 2.9, *p* < 0.05). More precisely, the gravitational and the inertial gains decreased and increased respectively when gravity increased. To conclude, a condition in which gravity itself could be manipulated–but not mass–also led to different gain adjustments, for very comparable ranges of LF.

## Discussion

In this experiment, we evaluated whether the mass and/or frequency of oscillations influence the control of GF as measured through a linear regression between GF and LF. We found that whatever the experimental condition, GF was always accurately predicted by a first order model although its parameters varied with mass and acceleration. Interestingly, we also showed that for a narrow interval of LF spanning most mass/frequency conditions, participants did not adjust GF equivalently, which may reveal high level mechanisms of GF control. A multiple regression model further showed that gravitational and inertial force components are treated independently according to the dynamical context.

### Global adjustments of the grip-load force coupling

The data confirm that participants modulate GF with LF with a high degree of precision in different loading conditions. Indeed, we found values of correlation coefficients compatible with other experiments (Flanagan et al., 1993). We characterized the modulation between the two forces with the gain and the offset. We did not find reliable effect of mass nor frequency on the gain. In contrast, Flanagan and colleagues (Flanagan and Rao, 1995) observed that increasing the average GF, either by increasing the frequency of oscillation or due to voluntary effort, can lead to a decrease in the slope. On the other hand, Zatsiorsky et al. (2005) reported that the slopes were steeper with heavier loads and decreased with movement frequency, for all masses. The present results seem contradictory regarding this point because (1) the slope was constant up to Mass 7 and (2) even increased for Mass 8. The two above studies suggest that the central controller might take into account, when determining the GF magnitude, not only the expected LF but also its origin and whether the force is increased due to the an increase of mass or acceleration. The discontinuity in the gain between Mass 7 and Mass 8 might be attributed to a psychological threshold linked to the mass, independently of frequency. In sum, this suggests that GF control is under high level mechanisms.

On the other hand, the data also indicate that mass and frequency contribute to increase the offset, in a non-linear fashion, as reported by the significant interaction between mass and frequency. This is consistent with the fact that when confronted with heavier loads and/or faster frequency, participants increased their average GF. This strategy is implemented to reduce the higher risk of dropping the object. Interestingly, the relative difference in offsets between the four frequencies increased with mass. Namely, doubling the mass or doubling the frequency did not result in the same shift in the average GF. This nicely demonstrates the power of internal models. Indeed, acceleration has a quadratic dependence on frequency and participants could well integrate that relation in the LF.

### Inertial and gravitational forces are processed independently by the CNS

In their interesting paradigm, Zatsiorsky and colleagues investigated the effects of the gravitational and inertial forces on GF by manipulating object mass and oscillation frequency. However, they did not compare how GF varied in a given range of LF.

We found that the CNS regulates GF differently if the same LF is generated by different combinations of masses and accelerations (see Figure 4). During slower movements, the gain decreased dramatically from 1.2 (lightest mass, Mass 2) to 0.4 (heaviest mass, Mass 8). The offsets in slow- and fast-frequency oscillations increased in parallel with mass, although being shifted upwards in rapid movements. It is interesting to ask why such differences exist in the control despite the similarity of LF. These results do not support the hypothesis that this low level—or automatic—modulation is implemented in order to optimize the sensitivity of the mechanoreceptors (Johansson and Westling, 1984, 1987; Edin, 2004). Since the same constraint was felt at the fingertips, it is unlikely that peripheral biological sensors might differentiate inertial from gravitational constraints (Angelaki et al., 2004). Therefore, there is strong indication that the analysis of the origin of LF must be situated at a higher level (Crevecoeur et al., 2009; Gaveau and Papaxanthis, 2011). This was further confirmed by the fact we measured different gravitational and inertial gains by altering mass, acceleration and gravity independently. This is in agreement with previous behavioral and brain imaging investigations that showed gravity is processed specifically in a wide panel of motor (Chang et al., 2000; McIntyre et al., 2001; Gentili et al., 2007) and visual tasks (Indovina et al., 2005; Zago et al., 2005; Senot et al., 2012). A sensory ambiguity also arises in identifying the actual motion associated with linear acceleration sensed by the otoliths in the inner ear (Fernández and Goldberg, 1976; Angelaki and Dickman, 2000). These internal linear accelerometers respond identically during translational motion and gravitational acceleration. Remarkably, Angelaki and colleagues identified motion-sensitive neurons in monkeys that provide a distributed solution to the ambiguous problem of differentiating inertial and gravitational accelerations as measured by the otoliths. This provides clear evidence that the dissociation is performed in the brain, and not at the periphery.

Our team had the opportunity to perform a similar experiment in parabolic flights, where gravity could be explicitly manipulated (White et al., 2005). We generated combinations of gravity (0, 1, 1.8 g), amplitudes of oscillations (20 or 40 cm) and masses of the test object (0.2 or 0.4 kg) so as to induce similar LF at the fingertips. The experimental constraints allowed an investigation of only a limited number of conditions. More importantly, the microgravity (0 g) condition did not overlap with a 1 or 1.8 g environment. However, data were sufficient to conclude at least qualitatively that inertial and gravitational forces are treated independently by the CNS. Another parabolic flight campaign allowed us to circumvent this limitation. When participants were asked to oscillate an object at a fast frequency across 0, 1, and 1.8 g, gravitational and inertial gains did not contribute equally to GF, as reported by our model that presented a better fit than when we did not really manipulate gravity. Although the range of LF overlapped almost totally across gravitational environments, we observed individual differences in GF profiles. Such behavior can be explained by personal aversions to risk of dropping the object (Westling and Johansson, 1984), different coefficients of friction of the fingertips (Cadoret and Smith, 1996) or other physical and psychological factors (Angst et al., 2010). In sum, this is in agreement with previous studies that reported that subjects are able to integrate the effects of gravity on LF when an object is held stationary (Hermsdörfer et al., 1999) or rhythmically moved vertically or horizontally (Hermsdörfer et al., 2000; Augurelle et al., 2003).

**Figure 7:**
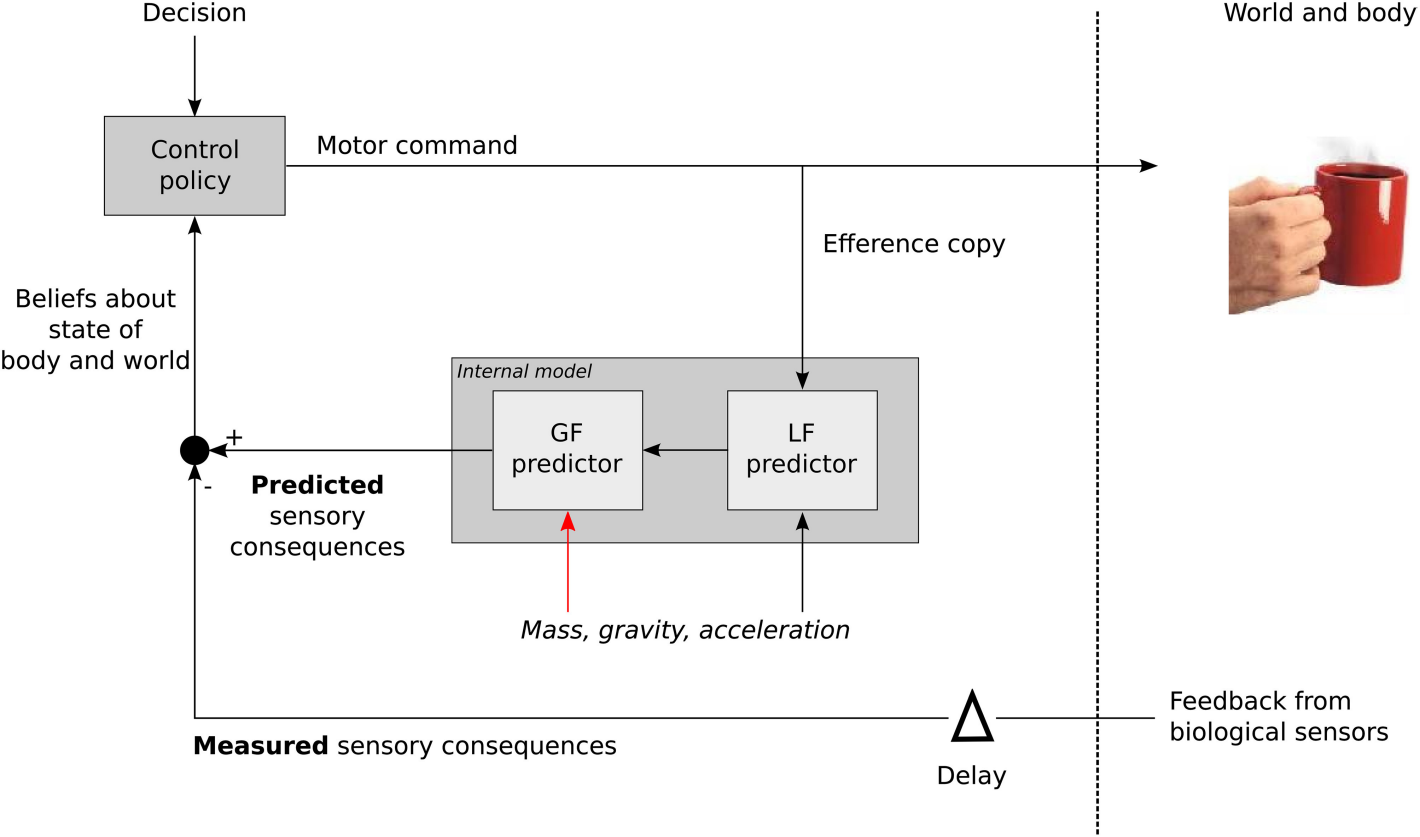
**General sketch illustrating how the contextual parameters mass, frequency (acceleration), and gravity are used to calculate GF when moving an object**. LF itself is not sufficient to derive GF that also need contextual information (red arrow).

This strengthens the existence of contextual internal models (Kawato, 1999; Davidson and Wolpert, 2004). Figure 7 describes in a simple formalism how the CNS treats the source of the load in order to generate the appropriate grip motor command. A control policy (inverse model) allows to derive the required motor commands in order to achieve a certain goal. An efference copy is then fed to an internal model that will first compute LF. Mass, gravity and acceleration are necessary to calculate LF but also GF (Figure 7, red arrow). In other words, the LF signal alone is not sufficient to compute a reliable GF. The gravitational and inertial gains set by that computational step may be tuned in order to provide an optimal response in case of perturbation for a specific context. The comparison between delayed biological feedbacks and predicted sensory consequences of the action allows to refine our beliefs about the state of the body and the world and to trigger any corrective action and update the relevant internal models. Higher level, more cognitive, adjustments are necessary to take into account the general characteristics of the environment itself. This is also in agreement with a previous study that suggested a high level of control to regulate the balance between inputs from a central pattern generator and resonant tuning in rhythmic movements, while mechanisms inherent to each of these modalities were of low level of control (White et al., 2008).

These results open up new questions in neurophysiology and robotics. Prehension is only mature at the age of 6 or 7 years old in children (Forssberg et al., 1991). Maturity translates as a smooth transition between feedback and feed forward processes (Blank et al., 2000; Smits-Engelsman et al., 2003). We therefore predict that young children may not be able to adjust their GF for the same composite LF but generated by different combinations of causes. Second, our investigations may influence the design of robotic grippers. Gravitational forces are defined by the external force field that exists independently of subject's actions. They are constant in magnitude and direction, and not subject to any change over time. In contrast, inertial forces are self-generated—therefore predictable to a certain extent—but variable. By treating these sources separately, a controller could adjust the inertial and gravitational gains more appropriately and optimize reactions to unexpected events that will likely affect the inertial forces only, leaving the gravitational component untouched. Therefore, the implementation of feedbacks could be optimized and the robustness of the system could be improved.

### Conflict of interest statement

The author declares that the research was conducted in the absence of any commercial or financial relationships that could be construed as a potential conflict of interest.
